# Study on the Dye Removal from Aqueous Solutions by Graphene-Based Adsorbents

**DOI:** 10.3390/ma16175754

**Published:** 2023-08-22

**Authors:** Paunka Vassileva, Vencislav Tumbalev, Diana Kichukova, Dimitrinka Voykova, Daniela Kovacheva, Ivanka Spassova

**Affiliations:** Institute of General and Inorganic Chemistry, Bulgarian Academy of Sciences, 1113 Sofia, Bulgaria; tumbalev@svr.igic.bas.bg (V.T.); diana123georgieva@gmail.com (D.K.); dimitrinka@svr.igic.bas.bg (D.V.); didka@svr.igic.bas.bg (D.K.); ispasova@svr.igic.bas.bg (I.S.)

**Keywords:** graphene oxide, adsorption, methylene blue, environment

## Abstract

In the current investigation, the removal efficiency regarding a cationic dye, methylene blue (MB), from three graphene-based materials was investigated. The materials’ characterization process involved instrumental methods such as XRD, XPS, SEM, TEM, FTIR, and nitrogen adsorption at 77 K. The survey examined how various process factors influenced the ability of the studied materials to adsorb cationic dyes. These parameters encompassed contact time, initial dye concentrations, solution pH, and temperature. The adsorption procedure was effectively explained through the application of pseudo-second-order and Langmuir models. The maximum adsorption capacity for the best adsorbent at 293 K was found to be 49.4 mg g^−1^. In addition, the study also determined the entropy, enthalpy, and Gibbs free energy values associated with the removal of MB and showed that the adsorption of MB is endothermic, feasible, and spontaneous. The results also revealed that the studied materials are suitable adsorbents for the removal of cationic dyes.

## 1. Introduction

Water pollution is a vital challenge facing humanity, given that wastewater poses serious risks to both the environment and human health [1]. Within this context, the treatment of dyed wastewater has garnered considerable interest. Dyes represent a significant group of synthetic organic compounds extensively utilized across multiple industries, particularly textiles. These compounds possess intricate aromatic molecular structures, rendering them highly stable and resistant to degradation. Persistence, non-biodegradability, and toxicity are some of the reasons why dyes have a harmful effect on water and soil in the environment [2,3,4]. Even at low concentrations (less than 1 ppm), their presence in water bodies leads to the abnormal penetration of sunlight and inflicts serious harm on aquatic organisms [5]. Various approaches have been performed for dye-cleaning in water, such as ion exchange, precipitation, osmosis, etc. [6]. However, due to the simplicity of the method and low cost, adsorption is most widely applied.

The basic dye, methylene blue (MB) (tetra-methylthionine chloride), finds extensive applications in the textile sector to dye materials such as cotton, silk, and wood [7,8]. It is also employed in microbiology, diagnostics, and surgery [9]. Although it is not extremely dangerous, it can have harmful effects on human health, causing eye burns, increased heart rate, diarrhea, jaundice, cyanosis, quadriplegia, shock, and tissue necrosis [4,9,10]. Hence, there is a crucial need to conduct research focused on eliminating this dye from water solutions. Methylene blue frequently serves as a representative compound for the removal of cationic dyes and other organic pollutants from aqueous solutions. In response to this challenge, researchers have been striving to create advanced materials capable of efficiently purifying water contaminated with MB.

Numerous studies have extensively examined the process of MB dye adsorption, employing a wide variety of adsorbents such as synthetic and natural polymers, inorganic materials, clays, and others [4,5,6,7,8,9,10,11,12,13,14,15,16,17,18,19]. Recently, attention has been directed to the application of carbon-based materials as adsorbents due to their abundance, structure, chemical stability, and suitability for practical application. Among these compounds, graphene-based nanomaterials, comprising graphene, graphene oxide (GO), and reduced graphene oxide (RGO), as newly developed carbon modifications have garnered considerable attention in scientific research, due to their unique structure and remarkable electronic and physicochemical properties [20,21,22]. Over the past few years, they have been applied as cutting-edge components in various scientific and technological domains, including nanomedicine, electronics, energy storage, power generation, etc. [23,24]. The unique structure of graphene provides the opportunity for all carbon atoms to be at the surface and both sides of the sheets are accessible for pollutant molecules. Graphene oxide is produced by the chemical oxidation process of graphite. This material possesses a specific two-dimensional atomic crystal structure, comprising a single layer of closely packed sp^2^-bonded carbon atoms arranged in a honeycomb lattice. Graphene oxide contains various types of functional groups on its surface (carboxyl, carbonyl, alkoxy, epoxide hydroxyl, and other oxygen-based functional groups) [25,26,27,28]. These oxygen-containing groups are responsible for the possibility of the surface functionalization of GO, which provides many opportunities for utilizing graphene oxide as a precursor in the synthesis of diverse nanocomposite materials. Reduced graphene oxide (RGO) is obtained by reducing the oxygen content of graphene oxide through thermal, chemical, or other approaches. This reduction process is carried out to enhance the honeycomb hexagonal lattice structure of the material [29]. Extensive research has demonstrated the potential of graphene family nanomaterials as effective nano-adsorbents. Their surface groups are involved in the adsorption processes. The mechanisms of adsorption have been identified as hydrogen bonding, electrostatic forces, π–π stacking, van der Waals forces, etc. [27,28]. Additionally, they act as weak acid cation exchange sites. Therefore, these materials have been widely reported as excellent adsorbents for the removal of diverse pollutants, including dyes, heavy metal ions, and radioactive substances [5,27,28,29,30,31,32]. Over the last decade, a variety of graphene-based materials, such as GO, RGO, and composite GO materials, have been effectively applied in the removal of methylene blue dye [1,2,5,7,9,27,28,33,34,35,36,37,38,39,40].

Data in the literature show a similar adsorption efficiency for both GO and RGO [41]. Many authors have reported that the hydrophilic character of GO may be a drawback for its practical use in the removal of dyes from aqueous media. They pointed out that GO disperses easily in water, which on the one hand, worsens its adsorption properties, and on the other leads to the difficult process of its separation from water. From this point of view, the RGO is a more appropriate adsorbent for practical use but possesses a lower number of surface functional groups that could embarrass the adsorption process.

For these reasons, the present study applied a new approach for handling the adsorption of MB from aqueous solutions; namely, the use of a monolith with a 3D-RGO hierarchical structure as an adsorbent. Its morphological and chemical uniformity allows for much easier use in practice than powders [42]. Moreover, the 3D-RGO adsorption properties can be further improved by additional surface functionalization.

In this present investigation, a 3D-hierarchical N-functionalized monolith was developed and investigated for its ability to remove the cationic dye methylene blue from water. The 3D-reduced graphene oxide monolith was successfully synthesized by the hydrothermal reduction of graphene oxide in the presence of ammonia. To evaluate its performance, it was compared with that of the starting material graphene oxide, and with that of reduced graphene oxide. Additionally, the study investigated the kinetics, thermodynamics, and desorption potential of methylene blue from the three materials.

## 2. Materials and Methods

### 2.1. Materials Preparation

**Graphene oxide.** GO was synthesized by a Tour method [43] from synthetic graphite (Sigma-Aldrich, Saint Louis, MO, USA, 99% carbon, 50 meshes), with the use of appropriate amounts of concentrated sulfuric acid, phosphoric acid, and potassium permanganate. The suspension was stirred for 1 h in an ice bath, followed by the addition of distilled water. The resulting material was washed to pH 6.5 with water. Then, by sonication of the GO for 1 h using the ultrasonic processor Sonix (Sonics & Materials, Inc., Newtown, CT, USA) (20 KHz, 750 W) the effective exfoliation to GO sheets was performed.

**Reduced graphene oxide.** RGO was obtained from the synthesized GO by reduction with L-ascorbic acid. Such prepared RGO was dried at 363 K for 12 h and thermally treated for 3 h at 873 K in Ar flow.

**Monolithic 3D-RGO.** The 3D graphene-based material was prepared by gelation of as-synthesized GO suspension under basic conditions. The aqueous GO suspension with a concentration of 25.5 mg.mL^−1^ was prepared by evaporation. In an autoclave equipped with a Teflon vessel, 8 mL of the GO suspension was mixed with 2 mL of concentrated NH_4_OH. The mixture was subjected to hydrothermal treatment at 433 K for 15 h in order to obtain a monolithic material. The preparation is based on a reduction process of GO to create self-assembled hierarchical porous structures, thus forming a macroscopic monolith. The obtained 3D graphene-based material was frozen in freeze-dry equipment the Alpha-Christ Freeze-Dryer (Christ, Osterode am Harz, Germany) for about 18–20 h using liquid nitrogen. The sample was denoted as M-RGO-N. 

### 2.2. Characterization

The phase composition of the materials was studied by powder X-ray diffraction (XRD). The diffractograms were collected within the 5–80° 2θ range on a Bruker D8-Advance Diffractometer (Karlsruhe, Germany) with CuK_α_ radiation and a LynxEye detector. The EVA software package supported by the ICDD-PDF-2(2021) database was applied for phase identification.

The morphology of the samples was studied by combining scanning electron microscopy (SEM) and transmission electron microscopy (TEM). SEM images were obtained on JEOL JSM-6390 (Tokyo, Japan) and TEM was performed on a TEM JEOL 2100 at 200 kV. Standard C/Cu grid was applied for loading the samples on it.

Low-temperature nitrogen adsorption at 77 K on Quantachrome Nova 1200e (Anton Paar Quanta Tech Inc., Boynton Beach, FL, USA) was performed for the determination of the texture parameters. A BET equation was used for the determination of the specific surface area; the total pore volumes and the mean pore diameters were calculated at p/p_0_ ≈ 0.99, according to the Gurvitch rule. The pore-size distributions were made by NLDFT (slit pores, equilibrium model).

The XPS measurements were performed on an AXIS Supra electron spectrometer (Kratos Analytical Ltd., Manchester, UK) using Al K_α_ radiation (1486.6 eV). The photoelectron spectra were analyzed in C1s, O1s, and N1s peak regions.

Fourier-transform infrared spectra (FTIR) of GO, RGO, and M-RGO-N in KBr were collected using a Thermo Nicolet Avatar 360 FTIR spectrometer (Thermo Fisher Scientific, Waltham, MA, USA), with a resolution of 2 cm^−1^.

### 2.3. Adsorption Studies

Adsorption tests were conducted in 50 mL conical flasks with a pre-weighed amount of 10 mg of adsorbent mixed with 10 mL of methylene blue dye solution. The mixtures were then subjected to shaking on a rotary shaker for varying periods. The solid particles were separated from the solution through centrifugation. The concentrations of methylene blue were analyzed using a Spekol 11 spectrophotometer (Carl Zeiss Industrielle Messtechnik GmbH, Jena, Germany), measuring the absorbance at λ_max_ = 650 nm. Batch adsorption experiments were conducted to examine the impact of contact time, pH, initial MB concentration, and temperature on the adsorption of methylene blue.

The equilibrium adsorption capacity (*Qe*, mg/g) was determined using the following equation:

*Q_e_* = (*C*_0_ − *C*) × *V*/*g* (1)

Here, *C*_0_ and *C*_e_ represent the initial and equilibrium concentrations of MB (mg L^−1^), respectively. *V* stands for the solution volume (L), and *m* corresponds to the mass of the sorbent (*g*). It is essential to note that each experiment was conducted in triplicate to ensure accuracy and reliability.

Methylene blue (MB) with a chemical formula of C_16_H_18_N_3_ClS and a molecular weight of 319.85 g mol^−1^ was procured from Merck (Darmstadt, Germany). To prepare a stock solution of MB dye with a concentration of 500 mg L^−1^, an appropriate quantity of dye powder was dissolved in deionized water (pH = 6.9). Subsequently, all the necessary working solutions with desired concentrations were prepared by diluting the stock solution with deionized water. This process allowed for the accurate preparation of solutions for the experiments. The adsorption behavior of methylene blue (MB) was investigated under various experimental conditions. To explore the impact of contact time, experiments were conducted at pH 6.9 with MB concentration of 50 mg L^−1^. The influence of the solution’s acidity on MB removal efficiency was examined over a pH range of 2.0 to 10.0, using an MB concentration of 50 mg L^−1^. To understand the effect of the initial MB concentration on adsorption capacity, different concentrations of MB ranging from 10 to 100 mg L^−1^ were tested at a pH of 8.0. Additionally, the influence of temperature was studied at 293, 303, and 333 K while maintaining a dye concentration of 100 mg L^−1^. Before mixing the suspensions, the pH of the initial solutions was carefully adjusted to the desired level using 0.1 M HCl and 0.1 M NaOH solutions. The desorption of MB was examined in a batch system using four different eluents: 2 M HCl, ethanol, water: ethanol (50:50), and 2 M HCl: ethanol (50:50). A weighed amount of the pre-adsorbed materials was added to 10 mL of each eluent and stirred for 24 h. The eluents were subsequently filtered and analyzed to determine the amount of desorbed methylene blue.

## 3. Results and Discussion

### 3.1. Materials Characterization

**Figure 1:**
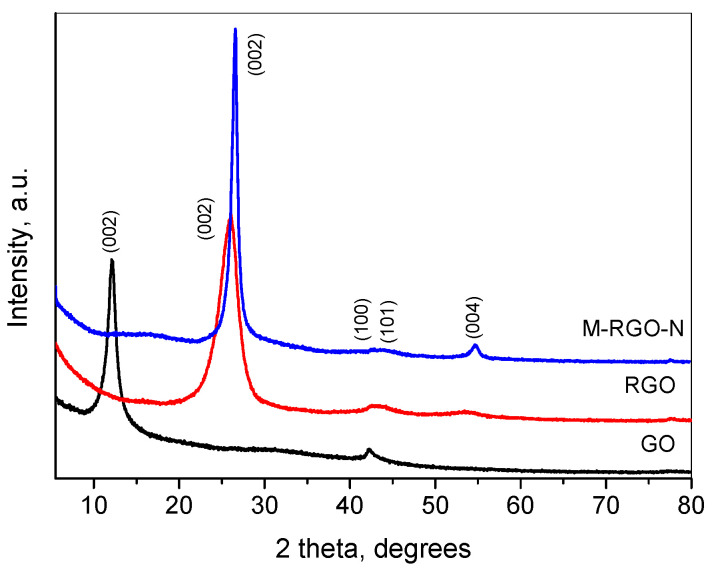
XRD patterns of GO, RGO, and M-RGO-N.

The XRD patterns of the GO, RGO, and M-RGO-N samples are presented in Figure 1. The pattern of GO comprises two main peaks—the first located at 12.3° 2θ (d = 7.1Å), and the second one at 42.4° 2θ (d = 2.1Å). The first peak is connected with the (002) interplanar distance of the graphite-type cell and reflects the increase of the **c** unit cell parameter perpendicular to graphite sheets. Such an increase is due to the intercalation of various molecular groups between the graphite sheets during the oxidation process. The FWHM of the (002) peak corresponds to a 8 nm size within this direction (≈10 graphene sheets). The second peak corresponds to the (100) plane of graphite. The patterns correspond to those already published in [28,39]. Upon reduction, the (002) peak shifts to the higher angle and for the RGO sample it is situated at 25.8° 2θ (d = 3.4 Å), similar to [34,36]. The FWHM of this peak reveals a 4 nm size within this direction, indicating a preserving of the number of stacked graphene sheets as in GO. As for the M-RGO-N pattern, the (002) peak is at 26.6° 2θ (d = 3.3Å) which corresponds to the reduced graphene oxide. The size within the <002> direction is about 13 nm (≈40 graphene sheets). The results show that the hydrothermal treatment led to the effective reduction of GO to RGO, although with an increased crystallite size.

**Figure 2:**
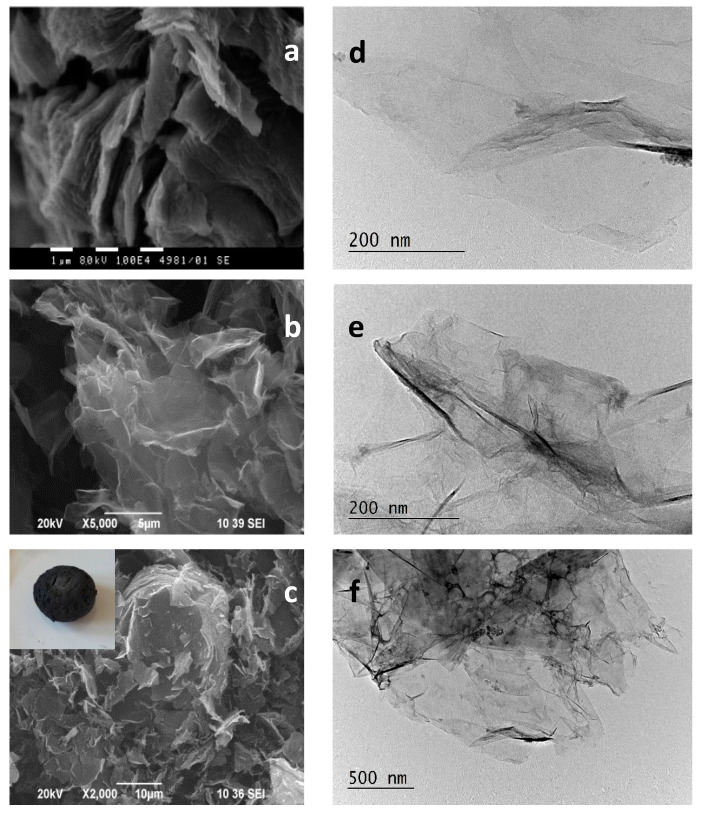
SEM (**left**) images of: (**a**) GO; (**b**) RGO, and (**c**) M-RGO-N and TEM (**right**) images of: (**d**) GO; (**e**) RGO, and (**f**) M-RGO-N.

The morphology of GO, RGO, and M-RGO-N combining SEM and TEM images of the corresponding samples is shown in Figure 2.

A sheet-like structure of all materials is clearly observed which reveals successful exfoliation of the initial graphite material. All samples consist of well-preserved graphene nanosheets, slightly rippled, and stacked in an arbitrary way, allowing the exposition of a large number of active sites for adsorption. The difference between them is in the size of the sheets and in the presence of a higher number of wrinkles and ripples in the reduced RGO and M-RGO-N samples. For the latter, the size of the sheets is the smallest and they are more crumpled, plicated, and broken. This fact is a prerequisite for increased specific surface area and pore volume.

**Figure 3:**
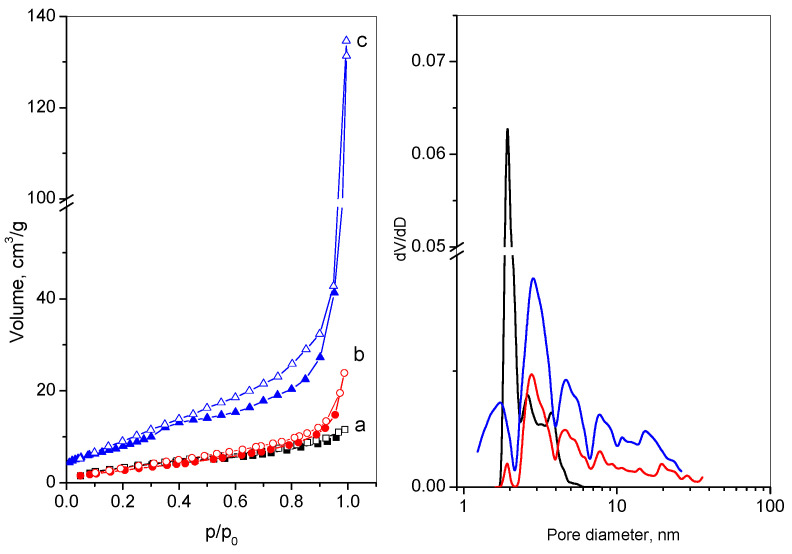
N_2_ adsorption–desorption isotherms (**left**) and pore-size distributions (**right**) of: (a) GO—black line; (b) RGO—red line; (c) M-RGO-N—blue line.

The N_2_ adsorption–desorption isotherms and the pore-size distribution of GO, RGO, and M-RGO-N are presented in Figure 3a,b, respectively. The texture parameters of the above-mentioned samples are placed in Table 1. GO and RGO exhibit II-type adsorption isotherms, according to the IUPAC classification, which is characteristic of a nonporous or macroporous material. The very narrow hysteresis is of type H3, which implies slit-shaped pores between the graphene sheets [44]. The isotherm of M-RGO-N shows a mixed-type isotherm—type I (attributed to micropores filling at low p/p_0_) and type II–pseudo II (connected with mesopores filling at high p/p_0_) [44]. The hysteresis shape H3 type reveals the non-rigid nature of the monolith, and the closure point of the adsorption and the desorption branches at about ≈0.4 p/p_0_ is consistent with the metastability of the condensate. The observed hysteresis loop is shifted to a higher p/p_0_ pressure and is an indication of the appearance of some macropores, as could also be seen from the pore-size distribution (Figure 3b). The pore-size distribution and the presence of a small volume of micropores (V_mi_, Table 1) are evidence for the formation of a hierarchical pore structure of the hydrothermally obtained M-RGO-N.

The texture parameters of GO and RGO are similar in their values.

The simultaneous process of GO reduction and N-modification during the hydrothermal synthesis leads to the enhanced specific surface area, total pore volume, and average pore diameter for M-RGO-N and is an indication of more successful exfoliation of the graphite. This confirms the results from XRD, SEM, and TEM. However, although freeze-drying was applied to restrict the effect of the capillary forces during the material’s drying, the obtained graphene sheets possess a relatively low surface area compared to the theoretical one, probably due to the incomplete exfoliation or sticking of the graphene sheets.

**Figure 4:**
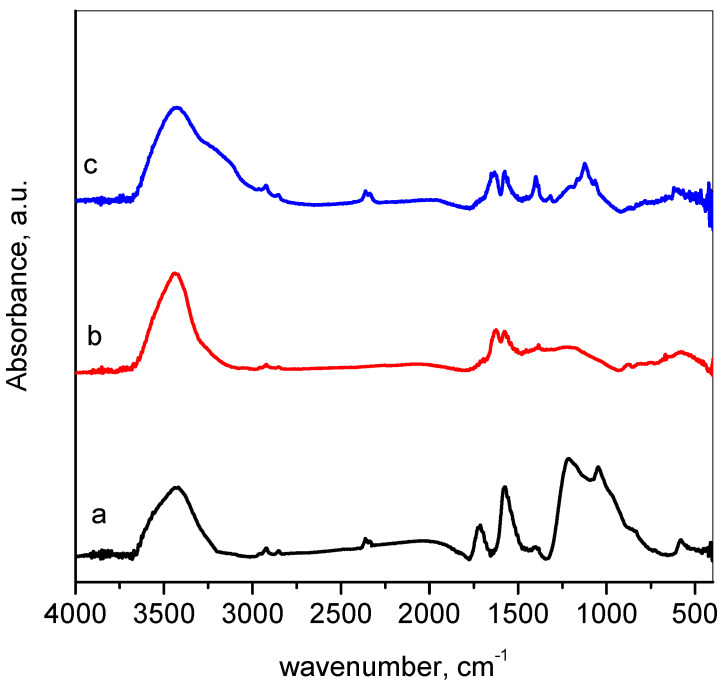
FTIR spectra of: (a) GO—black line; (b) RGO—red line; (c) M-RGO-N—blue line.

Figure 4 presents the Fourier-transform infrared spectra of the investigated materials. The GO spectrum (Figure 4a) consists of broadband at the region 3200–3600 cm^−1^ assigned to stretching vibrations of O–H. The bands at 2925 and 2854 cm^−1^ could be due to the C–H stretching vibrations [45]. The next bands at 1730 cm^−1^ and 1574 cm^−1^ are attributed to C=O and C=C in aromatic rings, respectively. Some overlapping bands form a broad band in the region 1400–890 cm^−1^, which should be a reflectance of the coexisting vibrations of epoxy, carboxyl, and carbonyl groups [46]. This band appears with decreased intensity in the spectrum of RGO, which indicates a successful reduction. The reduction also led to the disappearance of the band at 1730 cm^−1^ (C=O) and the appearance of a band at 1630 cm^−1^, assigned to the C=C bond. The spectrum of M-RGO-N shows an additional hump at about 3300–3100 cm^−1^ assigned to the stretching vibrations of N–H bonds. The intense band in 1285–1030 cm^−1^ indicates, similar to GO, the presence of oxygen-containing as well as nitrogen-containing groups [47].

**Figure 5:**
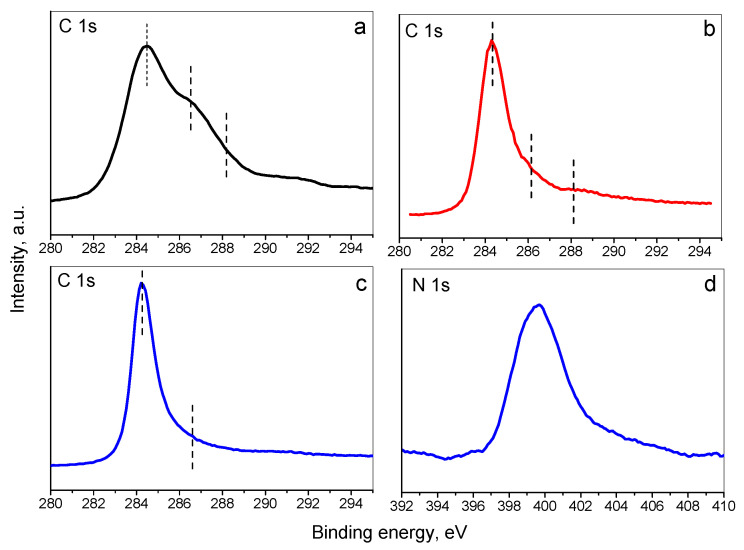
XPS photoelectron spectra of: (**a**) C1s of GO; (**b**) C1s of RGO; (**c**) C1s of M-RGO-N; (**d**) N1s of M-RGO-N.

XPS was performed in order to investigate the surface of the synthesized adsorbents. Figure 5a–c presents the C1s spectra of GO, RGO, and M-RGO-N, and the N1s spectrum of M-RGO-N, respectively. It is evident, that the main peak in the C1 spectrum of GO is situated at 284.5 eV and could be attributed to the C=C sp^2^ graphitic carbon bonds. This XPS peak is wide and asymmetric with shoulders at higher binding energies, suggesting the presence of sp^3^ carbon and oxygen-containing functional groups. The XPS C1s spectra for RGO and M-RGO-N are similar, with peaks with binding energy at 284.3 eV, less asymmetric than the peak for GO, which could be related to the reduction of the oxygen-containing groups. The N1s spectrum for M-RGO-N evidences the successful incorporation of nitrogen atoms during the hydrothermal production of the monolith and the surface nitrogen concentration was determined to be 4.5 at.%. The N1s peak is centered at 399.8 eV, but is asymmetric, suggesting a presence of nitrogen atoms in pyridinic, pyrrolic, or amine species [48].

### 3.2. Adsorption Studies

#### 3.2.1. Effect of pH on the Adsorption Process

**Figure 6:**
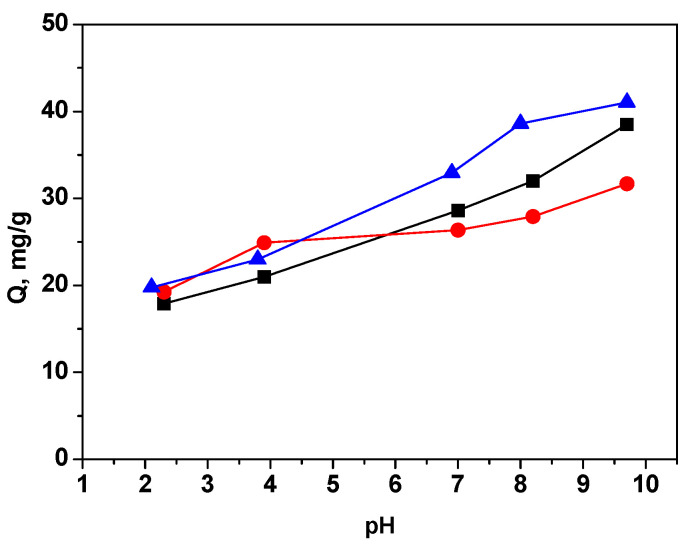
Effect of pH on MB adsorption by GO (black), RGO (red), and M-RGO-N (blue).

The pH of the aqueous solution affects the adsorption of dyes by changing the surface charge and ionization of the adsorbent as well as the dye. The regulation of electrostatic interactions between the adsorbent and the adsorbate plays a pivotal role in this context. To explore the impact of the initial solution’s pH on the adsorption of MB by the investigated materials, experiments were conducted across a pH range from 2.0 to 10.0. The outcomes of these experiments are depicted in Figure 6.

It is seen that the adsorption steadily increased with increasing the solution pH on the three sorbents. At low pH values, the surface of the adsorbents was positively charged (due to protonation of COOH, OH, etc. groups). Therefore, repulsion occurs between the surface of the studied samples and the cationic dye molecules, resulting in lower adsorption. At higher pH values, an improvement in the removal capacity of MB is seen. The increase in adsorption with increasing pH is due to the deprotonation of the functional groups present on the surface of the GO-based materials. It generates the negative charges on the surface of the adsorbents that attract the dye molecules, the electrostatic attraction becoming stronger as the pH of a solution increases. Additionally, the presence of liberated H^+^ ions can be reduced through a neutralization process, which, in turn, enhances the adsorption of MB [49]. The maximum adsorption performance of MB at pH 10 and higher was attained.

The final pH (pH_f_) of the adsorption solution is evaluated to calculate ΔpH (pH_f_—pH_i_). ΔpH was plotted against pH_i_ to determine the point of zero charge (PZC) associated with the investigated materials. The pH_pzc_ of GO, RGO, and M-RGO-N was observed to be around 2.8, 3.0, and 4.8, respectively. It is evident that these three materials exhibit a negative charge, even when placed in an acidic environment.

#### 3.2.2. Effect of Contact Time and Kinetic Study

**Figure 7:**
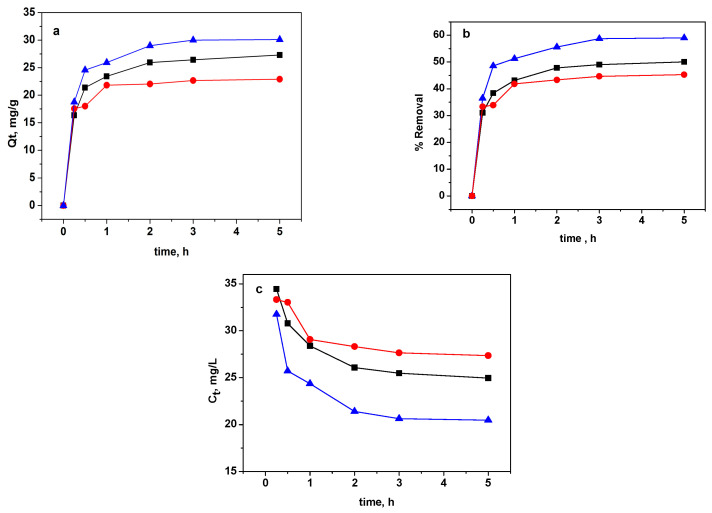
Plots of the adsorbed amount (**a**), % removal (**b**), and concentration (**c**) of MB as a function of time: for GO (black), RGO (red), and M-RGO-N (blue).

The impact of contact time on the MB removal by GO, RGO, and M-RGO-N was explored throughout a 5 h period. Figure 7a–c shows the adsorbed amount, % removal, and concentration of MB as a function of contact time, respectively. As the agitation time increased, the adsorbed amount and % removal of MB (according to [50]) showed a corresponding increase, reaching its maximum value within 120 min for both GO and M-RGO-N. With RGO, the dye adsorption required less time to reach equilibrium—60 min. As can be seen from Figure 7c, the largest decrease in the initial MB concentration upon reaching equilibrium is achieved with the M-RGO-N material. Nevertheless, these results suggest a good affinity of the three investigated sorbents for MB. All subsequent experiments were conducted with a contact time of 5 h.

The adsorption mechanism can be elucidated using various models. Among them, the commonly employed models for investigating adsorption kinetics include the pseudo-first-order, pseudo-second-order kinetic models, and intraparticle diffusion models.

*log*(*Q_e_* − *Q_t_*) = *log*(*Q_e_*) − (*k*_1_/2.303)*t* (2)

(*t*/*Q_t_*) = (1/*k*_2_*Q_e_*) + (1/*Q_e_*)*t* (3)

*Q_t_* = *k_id_ t*^1/2^ + *C* (4)

**Figure 8:**
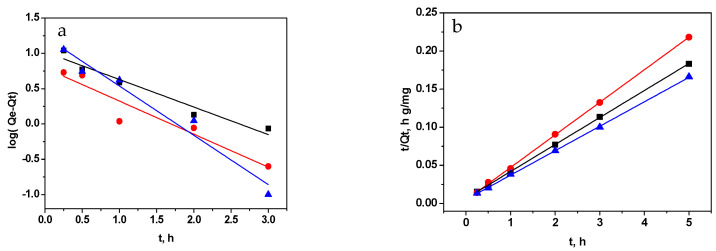

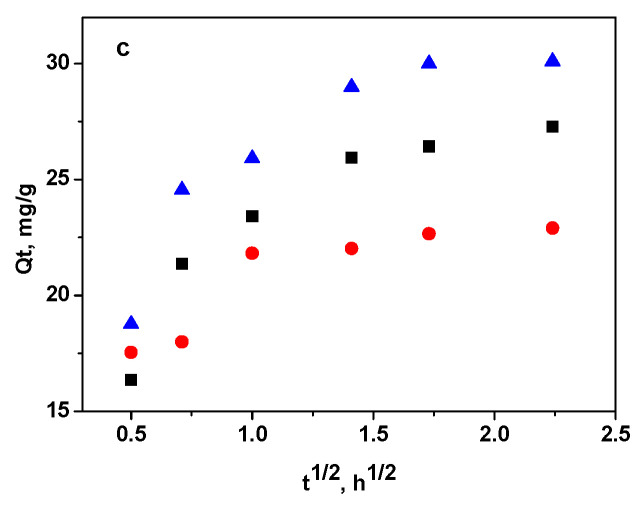
Application of (**a**) pseudo-first-order model, (**b**) pseudo-second-order model, and (**c**) intraparticle diffusion model for the adsorption of MB by GO (black), RGO (red), and M-RGO-N (blue).

Here, *Q_t_* (mg g^−1^) signifies the adsorbed amount of MB at a specific time t, *Q_e_* (mg g^−1^) represents the equilibrium adsorption capacity, *k*_1_ (h^−1^), *k*_2_ (g mg^−1^h^−1^) and *k_id_* (mg g^−1^h^−1/2^) represent the rate constant of pseudo-first-order adsorption, pseudo-second-order adsorption and intraparticle diffusion constant, respectively. Additionally, the values of k_1_ were determined from the slope of the plots *log*(*Q_e_* − *Q_t_*) versus *t* (Figure 8a), *k*_2_ values were calculated from the slope of the plots *t*/*Q_t_* versus *t* (Figure 8b), and *k_id_* values were derived from the slope of the plots *Q_t_* versus *t*^1/2^ (Figure 8c). The intercepts of the curves from the adsorption models were utilized to determine the essential parameter: equilibrium capacity (*Q_e_* and *C*). The correlation coefficients (r^2^-values), rate constants, and other relevant parameters for the three kinetic models (pseudo-first-order, pseudo-second-order, and intraparticle diffusion) were computed and are presented in Table 2.

The adsorption of methylene blue on the three studied materials exhibits kinetics that closely resemble a second-order process. The correlation coefficients calculated for this model were found to be very close to unity, indicating a strong agreement with the experimental data. Furthermore, the equilibrium capacities estimated from the pseudo-second-order model showed a strong correlation with the experimentally observed *Q_e_* values. The findings indicate that the MB adsorption process can be effectively described by the pseudo-second-order kinetic equation. These observations suggest that the rate of adsorption reaction is affected by two main factors: the concentration of MB in the solution and the abundance of available adsorption sites on the surfaces of the adsorbents [51]. Moreover, the adsorption behavior of methylene blue for all the studied materials is predominantly governed by chemical-controlling processes.

To assess the significance of diffusion as a controlling process in determining the rate of MB adsorption on GO, RGO, and M-RGO-N, the diffusion kinetic equation proposed by Weber and Morris was also employed. The findings from the kinetic analysis suggest that the adsorption process involves multiple stages. The kinetics plot exhibits two distinct linear regions, implying a complex adsorption mechanism. However, the results clearly indicate that diffusion does not act as the rate-determining step during MB adsorption in this particular case.

#### 3.2.3. Adsorption Isotherms

**Figure 9:**
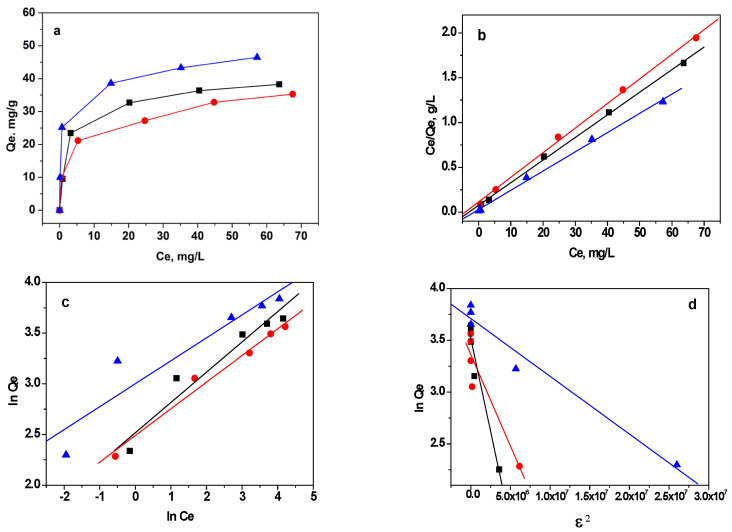
(**a**) Experimental adsorption isotherms and the application of (**b**) Langmuir, (**c**) Freundlich and (**d**) Dubinin–Radushkevich isotherms to the adsorption of MB on GO (black), RGO (red), and M-RGO-N (blue).

To investigate the impact of the initial concentration of MB on the adsorption capacities of the studied GO-based materials, a range of MB concentrations from 10 to 100 mg L^−1^ was used at pH 8.0 (as shown in Figure 9a). pH 8 was chosen for the study because at high pH values, the molecular structure of MB can undergo changes due to stepwise demethylation [33]. The adsorption isotherm provides valuable insights into the distribution of adsorbate molecules or ions between the solid adsorbent and the solution at equilibrium. It describes how the quantity of adsorbate on the adsorbent’s surface relates to its concentration in the solution, under specific conditions such as temperature and concentration. Different types of model adsorption isotherms help in understanding the adsorption process and characterizing the adsorbent’s behavior in terms of its capacity and affinity for the adsorbate. In the present study, MB equilibrium data were analyzed using three widely used isothermal models: the Langmuir, Freundlich, and Dubinin–Radushkevich models, whose equations are formulated based on different principles, as described in [51]. The Langmuir model hypothesizes that adsorption occurs at uniform and homogenous sites within the adsorbent, making it suitable for describing monolayer adsorption processes. On the other hand, the Freundlich model suggests that the adsorbates are taken up on a heterogeneous surface through multi-layer adsorption. The Dubinin–Radushkevich isotherm, in contrast, sheds light on the adsorption mechanism by employing potential theory to explain the adsorption process. The compatibility of the experimental data with a particular equation provides insight into the probable mechanism of adsorption. The linear graphs for the three isotherm models are presented in Figure 9b–d. The calculated values for isotherm constants and correlation coefficients are given in Table 3. The data for the adsorption of MB on the three GO-based materials were well fitted by the Langmuir isotherm model (Figure 9b), as demonstrated by the high correlation coefficients (r^2^ greater than 0.996), compared to those for the Freundlich, and Dubinin–Radushkevich (D-R) isotherm models. These findings suggest that the predominant mechanism of MB adsorption involves binding to a relatively uniform surface and the formation of a monolayer.

GO shows a relatively higher MB adsorption capacity compared to RGO because the surface of GO comprises more oxygen-containing functional groups and therefore, more adsorption sites than in RGO. The M-RGO-N material shows the highest adsorption capacity towards MB. This could be explained by the texture parameters (specific surface areas, pore volumes) and the presence of hierarchical pores, as well as the surface functionalization with nitrogen-containing groups that may play the role of additional active centers for dye adsorption. Thus, the combination of its porous structure and surface functionalization with nitrogen groups makes M-RGO-N a highly efficient adsorbent for MB.

The capacities towards MB obtained in the present study are compared to other adsorbents based on GO reported in the literature (Table 4) [28,34,35,36,37,38,39,40,49,52,53,54,55]. It could be clearly seen that the maximum Langmuir capacity varies very significantly, depending on the type of material, the conditions of the experiment, and the mechanism of adsorption. It is worth mentioning that the adsorption capacities for MB of the materials in the present study display comparable values to the data presented in the literature, which makes the investigated graphene-based materials appropriate for MB removal from aqueous solutions.

The n-values obtained from the Freundlich isotherm model (Figure 9c) for GO, RGO, and M-RGO are 3.34, 3.80, and 4.41, respectively [51]. These values point to a favorable adsorption process for all three materials, as higher n-values signify stronger adsorption. On the other hand, the Dubinin–Radushkevich isotherm model provides insights into the adsorption energy. The calculated mean free energy values for GO, RGO, and M-RGO-N are 1.20, 1.74, and 3.78 kJ mol^−1^, respectively. These values suggest that the predominant adsorption mechanism is physisorption.

#### 3.2.4. Thermodynamic Studies

The thermodynamic parameters of adsorption from solutions are crucial in providing valuable insights into the type and mechanism of the adsorption process. They offer information about the driving forces that govern the interaction between the adsorbate and the adsorbent. By studying these thermodynamic parameters, researchers can determine the feasibility, spontaneity, and nature of the adsorption process, which is essential for optimizing and understanding the various practical applications of adsorption in different fields. To investigate the effect of temperature on MB removal, experiments were conducted at different temperatures: 293 K, 303 K, and 333 K. As the temperature increased, the adsorbed amounts of MB on each adsorbent also increased. This observation suggests that the adsorption process is characterized as endothermic.

The changes in Gibbs free energy (Δ*G*^0^), enthalpy (Δ*H*^0^), and entropy (Δ*S*^0^) were calculated using the following equations:

*K_d_* = *Q_e_*/*C_e_* (5)

Δ*G*^0^ = −*RT lnK_d_* (6)

*lnK_d_* = Δ*S*^0^/*R* − Δ*H*^0^/*RT* (7)

**Figure 10:**
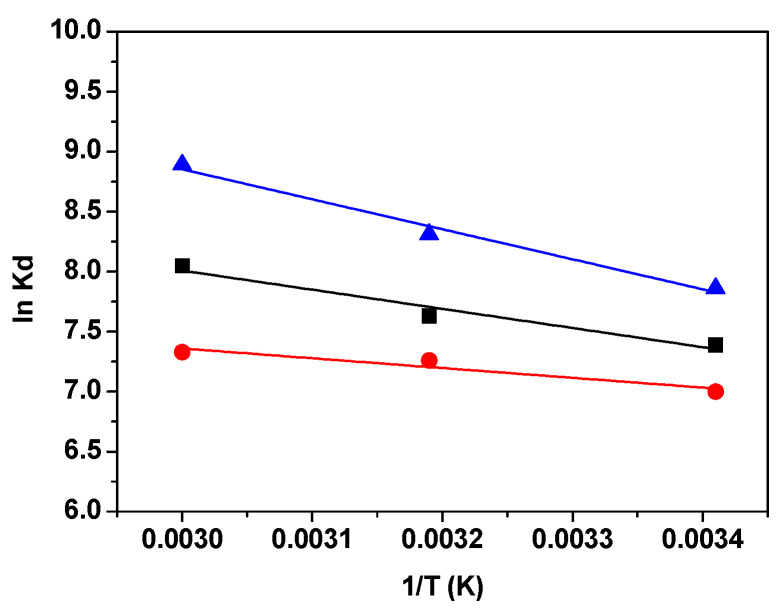
The relationship between the equilibrium constant (*K_d_*) and temperature: GO (black), RGO (red), and M-RGO-N (blue).

In the equations provided, *K_d_* represents the equilibrium constant, *R* is the gas constant (measured in J mol^−1^K^−1^), and *T* stands for the temperature (in Kelvin). By plotting *lnK_d_* against 1/*T*, we can determine the enthalpy change, Δ*H*^0^, from the slope of the graph, and the entropy change, Δ*S*^0^, from its intercept (as shown in Figure 10). The values of the thermodynamic parameters Δ*G*^0^, Δ*H*^0^, and Δ*S*^0^ are compiled and presented in Table 5.

Changes in the standard free energy at different temperatures have negative values, indicating that the adsorption of MB dye on the materials GO, RGO, and M-RGO-N is feasible and spontaneous. The Δ*G*^0^ value for the MB−M-RGO-N system is more negative compared to the other two systems, suggesting more spontaneous adsorption of MB onto M-RGO-N. Generally, Δ*G*^0^ values for physisorption range between 0 and 20 kJ mol^−1^, while chemisorption is between 80 and 400 kJ mol^−1^ [56]. The calculated Δ*G*^0^ values in the present study reveal that physisorption is the dominating mechanism. For the MB−M-RGO-N system, Δ*G*^0^ values are in the range −19.03−24.55 kJ mol^−1^, which indicated a more complex adsorption mechanism. On the other hand, the positive values of Δ*H*^0^ (in the range 7.28–21.41 kJ mol^−1^) confirmed that the adsorption was mainly physical and the fact that the adsorption efficiency increased with the temperature increase. The positive values of the entropy change (Δ*S*^0^) correspond to a decrease in the molecular orderliness at the interface between the solid and liquid phases during the adsorption process, leading to an increase in the degree of freedom of the adsorbed dye molecules. The positive value of Δ*S*^0^ may indicate the irreversibility and stability of the adsorption [38,57]. The observed small positive values of Δ*S*^0^ in the studied systems suggest that there are no significant structural changes in the surfaces of the adsorbents during the adsorption process.

Adsorbent regeneration is a problem of great importance. Therefore, the desorption capability of MB from the studied adsorbents was examined. In the present study, batch desorption experiments were conducted using different solvents, including 2 M HCl, ethanol, water: ethanol (50:50), and 2 M HCl: ethanol (50:50). However, the desorption efficiency was found to be remarkably low. The highest percentage of MB desorption was achieved using a water: ethanol mixture, with values of 18% for GO, 6% for RGO, and 14% for M-RGO-N, respectively. The limited desorption percentage can be attributed to the special structure of the studied materials. Due to the small size of the MB molecule, it easily becomes adsorbed and forms a stable complex with the adsorbent, making the desorption process highly difficult [58]. On the other hand, this outcome could be attributed to a more intricate combined adsorption mechanism, a mixed physisorption–chemisorption process. It is possible that not only electrostatic interactions but also π–π interactions between the aromatic rings of MB and graphene oxide-based materials as well as hydrogen bonding are involved in the adsorption process [31].

## 4. Conclusions

The removal efficiency of three graphene-based materials for the adsorption of methylene blue from water was studied. The graphene materials—graphene oxide, reduced graphene oxide, and novel 3D-reduced graphene oxide monolith—were successfully prepared via the modified Tour method, reduction with ascorbic acid, and hydrothermal reduction in the presence of ammonia, respectively. The results from various characterization techniques revealed in detail the structural, morphological, and surface characteristics of the adsorbents. The prepared monolith was confirmed to be 3D-hierarchical and nitrogen functionalized. The influence of main process factors (contact time, initial dye concentrations, solution pH, and temperature) on the ability of these materials to adsorb cationic dyes was studied. The adsorption procedure was effectively explained through the application of pseudo-second-order and Langmuir models. The determined entropy, enthalpy, and Gibbs free energy values for the removal of MB showed that the adsorption of MB is feasible, spontaneous, and endothermic. The highest adsorption capacity was found to be exhibited by a 3D-reduced monolith of graphene oxide—49.4 mg g^−1^ at 293 K. The combination of its porous structure and surface functionalization with nitrogen groups makes M-RGO-N an efficient adsorbent for MB; moreover, it can be easily separated from the solution. Nevertheless, the results established that the three studied materials are suitable adsorbents for the removal of cationic dyes.

## Figures and Tables

**Table 1:** Texture characteristics of GO, RGO, and M-RGO-N.

Sample	S ^1^ m^2^/g	V ^2^ cm^3^/g	D_av_ ^3^ nm	V ^4^_mi_ cm^3^/g
GO	14	0.02	5	-
RGO	18	0.05	12	-
M-RGO-N	40	0.18	18	0.02

^1^ BET; ^2,3^ at p/p_0_ ≈ 0.99; ^4^—Dubinin–Radushkevich method.

**Table 2:** Kinetic parameters for MB adsorption.

Sample	Pseudo-First-Order Constants			Pseudo-Second-Order Constants			Intraparticle Diffusion Constants		
	*Q_e_* (mg g^−1^)	*k*_1_ (h^−1^)	r^2^	*Q_e_* (mg g^−1^)	*k*_2_ (g mg^−1^h^−1^)	r^2^	*k_id_* (mg g^−1^ h^−1/2^)	*C* (mg g^−1^)	r^2^
GO	10.46	0.169	0.9399	28.24	5.531	0.9996	5.650	16.33	0.8695
RGO	6.19	0.203	0.8820	23.41	8.173	0.9997	3.499	16.02	0.8728
M-RGO-N	17.18	0.303	0.9545	31.15	6.275	0.9996	5.912	11.12	0.8808

**Table 3:** Isotherm constants and correlation coefficients for MB adsorption.

Sample	Langmuir Parameters ^1^			Freundlich Parameters ^2^			D-R Parameters ^3^		
	*Q*_0_ (mg g^−1^)	*K*_1_ (L mg^−1^)	r^2^	*k_F_* (mg^1−n^L^n^ g^−1^)	*n* (L mg^−1^)	r^2^	*Q_m_* (mg g^−1^)	*E* (kJ mol^−1^)	r^2^
GO	39.81	0.335	0.9984	12.39	3.344	0.9546	32.46	1.20	0.9352
RGO	36.63	0.241	0.9977	12.07	3.802	0.9512	27.99	1.74	0.8227
M-RGO-N	49.38	0.711	0.9958	20.09	4.415	0.9508	33.32	3.78	0.9567

^1^*C_e_*/*Q_e_* = 1/*K_L_Q*_0_ + *C_e_*/*Q*_0_; ^2^ *lnQ_e_* = ln*k_F_* +(1/*n*) *lnC_e_*; ^3^ *lnQ_e_* = *lnQ_m_* − *βε*^2^, *ε* = *RTln*(1 + 1/*C_e_*), *E* = 1/(−2*β*)^1/2^.

**Table 4:** Comparison of the values of the adsorption capacities of graphene-based materials with respect to MB reported in the literature.

Adsorbent	*Q_max_* (mg/g)	Conditions	Reference/ Year
Monolayer GO	440.5−803.7	pH = 3, 7, 11; 2.35 mg GO; MB range: 0.05–0.475 g/L; 298 K	[28] (2022)
rGO	81	283 K, 10–50 mg/L, 0.01 g adsorbent	[34] (2013)
GO	119.0	293 K, 180 min, pH = 4, 600 mgL^−1^	[35] (2021)
rGO	64.8 and 80.1	MB 20−50 mgL−1 dosage of 50 mgL^−1^; pH = 4	[36] (2019)
GO	429.5	5–25 ppm MB, 10 mg adsorbent, 313 K, 15 min, pH 8	[37] (2019)
rGO	121.9	pH = 6, 298 K, 10–100 mg/L MB, 200 mg adsorbent^−1^	[38] (2020)
GO	311.7	6–100 ppm MB, 0.5 gL^−1^ adsorbent, 293 K, pH = 10	[39] (2021)
GO-CS composites	7.5	MB 10 mg/L, pH = 5, adsorbent 0.143 g/L, 125 min	[40](2022)
Chitosan-Functionalized GO	23.3	MB 10−50 mg/L, 35 min, GO@CS 50 mg/10 mL, pH = 7, 25 °C	[49] (2022)
rGO/TNT	26.3	MB 1−60 mg/L, 25 °C, 0.1 g/10 mL adsorbent, pH 6.8	[52] (2019)
GO/polyaniline	14.2	MB 10−80 mg/L, 270 min, 0.3–0.4 g adsorbent, pH = 6.5	[53] (2019)
rGO/polyaniline	19.2		
GO	48.8–598.8	298 K, pH = 7, 0.015 g GO, MB 5–600 mg dm^−3^	[54] (2014)
GO	17.3	MB 10−50 mg/L, 25 °C, 0.22 mg/mL adsorbent	[55] (2011)
GO	39.8	MB 10−100 mg/L, 293 K, pH = 8, 0, 10 mg adsorbent	Present study
RGO	36.6		
M-RGO-N	49.4		

**Table 5:** Values of *ΔG^0^*, *ΔH^0^*, and *ΔS^0^* at different temperatures for MB adsorption.

Adsorbent	Δ*H*^0^_,_ kJ mol^−1^	Δ*S*^0^, J mol^−1^ K^−1^	Δ*G*^0^, kJ mol^−1^		
			293 K	313 K	333 K
GO	13.72	107.82	−17.87	−18.95	−22.18
RGO	7.28	83.14	−17.05	−17.91	−20.36
M-RGO-N	21.41	138.01	−19.03	−20.41	−24.55

## Data Availability

Data sharing is not applicable to this article.
